# A100 CAVERNOUS TRANSFORMATION OF THE PORTAL VEIN IN A PEDIATRIC PATIENT WITH GOLDENHAR SYNDROME: A CASE REPORT

**DOI:** 10.1093/jcag/gwac036.100

**Published:** 2023-03-07

**Authors:** F Diaz, F Briglia

**Affiliations:** Pediatrics and Child Health, University of Manitoba, Winnipeg, Canada

## Abstract

**Background:**

Goldenhar syndrome is a rare congenital condition thought to arise from the first and second branchial arches and typically asymmetrically affects the eyes, ears, and spine. Portal vein thrombosis/cavernous transformation is one of the main causes of portal hypertension in children. It is often associated with risk factors such as catheterization of the umbilical vein in the neonatal period, omphalitis, intra-abdominal infections in the neonatal period and prothrombotic conditions. This is a challenging clinical scenario as it leads to portal hypertension and variceal bleed with no chronic liver disease stigmata and liver function is essentially normal. While previous European literature from the early-2000s reported associated portal vein anomalies in Goldenhar syndrome, we are not aware of any recent and/or Canadian pediatric cases.

**Purpose:**

We report a Canadian case of cavernous transformation of the portal vein with resulting gastrointestinal bleeding in a child with Goldenhar syndrome.

**Method:**

Case report

**Result(s):**

A pediatric patient with a postnatal suspicion of Goldenhar syndrome, with confirmation by 6 months of age, presented with an acute 3-day history of melena, on the context of a recent viral illness and ibuprofen use. Laboratory testing showed a normocytic anemia (Hb 68) with a normal INR (1.1). The rest of the workup was unremarkable. There was no history of catheterization of the umbilical vein on the neonatal period nor other hematological complications. To assess for potential duplication cyst as differential diagnosis, a computerized tomography of the chest, abdomen and pelvis was ordered and revealed suspected cavernous transformation of the portal vein with suspected varices near the gastroesophageal junction and within the abdomen, and hepatomegaly with spleen size at the upper limit of normal. Octreotide was started after the CT results. A gastroscopy was done 24h after presentation and found portal hypertensive gastropathy and esophageal varices (2 Grade II and 1 Grade I). Sclerotherapy was performed. Post-gastroscopy ultrasound supported earlier radiographic findings of cavernous portal vein transformation and hepatosplenomegaly. Moreover, the ultrasound noted normal flow direction in the portal and hepatic veins. The patient has not re-presented for further episodes of gastrointestinal bleeding.

**Conclusion(s):**

This case report supports the previously reported association between Goldenhar syndrome and portal venous anomalies. Early consideration may lead to prompt diagnosis and management of the potentially life-threatening complications of portal hypertension in this population.

**Please acknowledge all funding agencies by checking the applicable boxes below:**

None

**Disclosure of Interest:**

None Declared

